# The immune microenvironment in non‐small cell lung cancer is predictive of prognosis after surgery

**DOI:** 10.1002/1878-0261.12475

**Published:** 2019-04-10

**Authors:** Åsa Kristina Öjlert, Ann Rita Halvorsen, Daniel Nebdal, Marius Lund‐Iversen, Steinar Solberg, Odd Terje Brustugun, Ole Christian Lingjærde, Åslaug Helland

**Affiliations:** ^1^ Department of Cancer Genetics Institute for Cancer Research Oslo University Hospital The Norwegian Radium Hospital Norway; ^2^ Department of Pathology Oslo University Hospital The Norwegian Radium Hospital Norway; ^3^ Department of Cardiothoracic Surgery Oslo University Hospital, Rikshospitalet Norway; ^4^ Department of Informatics University of Oslo Norway; ^5^ Department of Clinical Medicine University of Oslo Norway

**Keywords:** gene expression, immune microenvironment, non‐small cell lung cancer, PD‐L1, prognosis, TP53

## Abstract

The impact of the tumor immune microenvironment on overall survival in non‐small cell lung cancer (NSCLC) has been studied, but there is little information on its relevance for risk of relapse after surgery. Understanding more about the immune microenvironment in previously untreated NSCLC could help in identifying high‐risk patients and patients more likely to benefit from neoadjuvant/adjuvant immunotherapy. Here, we examined gene expression in 399 surgically derived NSCLC samples and 47 samples from normal lung, using Agilent microarray and RNA sequencing. In 335 of the tumor samples, programmed death‐ligand 1 (PD‐L1) expression was evaluated by immunohistochemistry. Gene expression was used to estimate content of immune cells and to calculate an immune score. Properties of the immune microenvironment, and its impact on prognosis, were compared in histological subgroups and gene expression subtypes. Tumors with an active immune microenvironment were found for both adenocarcinomas (AD) and squamous cell carcinomas (SCC). In AD, high immune score and high estimates of several immune cell types belonging to the adaptive immune system were associated with better progression‐free survival (PFS), while in SCC, no association between immune characteristics and PFS was found. The immune microenvironment, including PD‐L1 expression, and its impact on prognosis showed clear differences in AD and SCC gene expression subtypes. In conclusion, the NSCLC immune microenvironment is predictive of prognosis after surgery. Lung AD and SCC gene expression subtypes should be investigated as potential prognostic biomarkers in patients treated with immune checkpoint inhibitors.

**Abbreviations**
aDC: activated dendritic cells
CD4+ Tcm: CD4+ central memory T cells
CD4+ Tem: CD4+ effector memory T cells
CD8+ Tcm: CD8+ central memory T cells
CD8+ Tem: CD8+ effector memory T cells
cDC: conventional dendritic cells
DC: dendritic cells
iDC: immature dendritic cells
NKT: natural killer T cells
pDC: plasmacytoid dendritic cells
PI: proximal inflammatory
PP: proximal proliferative
Tgd cells: gamma delta T cells
Th1 cells: type 1 T‐helper cells
Th2 cells: type 2 T‐helper cells
Tregs: regulatory T cells
TRU: terminal respiratory unit

## 1 Introduction

Lung cancer is often diagnosed in advanced stage, and the 5‐year survival rate is 10–20% (Allemani *et al*., 2015). Patients with early stage non‐small cell lung cancer (NSCLC) can be offered surgery with curative intent, but many of these will later relapse. The most significant predictor for recurrence is TNM stage. Other clinical factors associated with poor prognosis are tumor grade (Sun *et al*., 2006), increasing age, male gender, smoking status, performance status, comorbidity, and surgical technique (Bugge *et al*., 2018; Wei *et al*., 2018; Woodard *et al*., 2016). Different prognostic biomarkers have been investigated but this far none have been enough validated to make its way to the clinic.

Since the role of the immune system was recognized in preventing and arresting the development of cancer, much effort has been put into understanding the underlying biology. Catacchio *et al*. reviewed the importance of different immune cell types for prognosis in lung cancer reporting M1 macrophages, CD8+ T cells, CD4+ T cells, mature dendritic cells (DC), and the presence of tertiary lymphoid structures in tumor to be associated with good prognosis. Regulatory T cells (Tregs), immature DC, and M2 macrophages were found to be associated with poor prognosis (Catacchio *et al*., 2018). A meta‐analysis on the prognostic impact of PD‐L1 expression in NSCLC showed poor prognosis in those with a PD‐L1‐positive tumor (Wang *et al*., 2015). Signs of systemic inflammation, for example, high neutrophil‐to‐lymphocyte ratio (Gu *et al*., 2015), have also been associated with poor overall survival (OS) and progression‐free survival (PFS).

In most studies, NSCLC has been investigated as one entity, although its main histological subgroups, adenocarcinomas (AD) and squamous cell carcinomas (SCC), in many ways behave differently. Both subgroups have high mutational burden, but they differ in mutational pattern. For example, activating *EGFR* mutations and *ALK* alterations are mostly found in AD while almost all SCC harbor a *TP53* mutation (Cancer Genome Atlas Research Network, 2012, 2014). The immune microenvironment shows different characteristics in AD and SCC (Socinski *et al*., 2016), and it is likely that its impact on prognosis varies with histological subgroup.

In 2006 and 2010, expression subtypes were described in AD and SCC, respectively (Hayes *et al*., 2006; Wilkerson *et al*., 2010, 2012), and these are, to some degree, predictive of patient outcome. In AD, the terminal respiratory unit subtype (TRU, formerly bronchioid) has better prognosis after surgery and includes most nonsmokers and patients with *EGFR* mutations and *ALK* alterations. The proximal inflammatory subtype (PI, formerly squamoid) often harbors mutations in *TP53* and *NF1* and has high mutational burden and proliferation. The proximal proliferative subtype (PP, formerly magnoid) shows high expression of DNA repair genes, high proliferation and often mutations in *TP53*,* KRAS,* and *STK11*. In this subtype, we find more heavy smokers and higher chromosomal instability and hypermethylation, compared to in the other subtypes (Cancer Genome Atlas Research Network, 2014; Wilkerson *et al*., 2012). In SCC, four different subtypes are described. The primitive subtype is characterized by high proliferation, poor differentiation, *RB1* and *PTEN* alterations, and poor prognosis. The classical subtype shows typical alterations associated with heavy smoking as high expression of genes active in detoxification and high overall methylation and chromosomal instability. The basal subtype is usually well differentiated with high activity in genes active in cell adhesion and in the basement membrane, while the secretory subtype expresses many genes related to immunological activity and secretory functions (Cancer Genome Atlas Research Network, 2012; Wilkerson *et al*., 2010).

Faruki *et al*. (2017) investigated the immune microenvironment in AD and SCC expression subtypes and found that the TRU and PI subtypes in AD, and the secretory subtype in SCC, had more immunologically active tumor microenvironments. The influence of estimated immune markers on OS was investigated separately in the expression subtypes and was found to be most important for the AD PI subtype and the SCC primitive subtype, where higher estimates of several immune cell types were associated with better OS. Lung cancer patients treated with curative intent are known to have increased mortality from other causes than lung cancer (Bugge *et al*., 2018). Most studies on prognosis after surgery focus on OS while identifying factors influencing risk of recurrence would be more meaningful to select high‐risk patients eligible for adjuvant treatment. The impact of different immune characteristics on risk of relapse after surgery in expression subtypes has not yet been studied.

Immune checkpoint inhibitors are used to treat advanced NSCLC, and trials with adjuvant and neoadjuvant immunotherapy are in progress. There is still a lack of good prognostic biomarkers for response. PD‐L1 expression and high mutational burden/neoantigen load have emerged as the most promising ones, but the absence of these has not been able to predict lack of response (Ahmadzada *et al*., 2018). The aim of this study was to achieve more knowledge about the immune microenvironment in NSCLC and how it influences risk of relapse after surgery, with the hope that this could help selecting patients for adjuvant immunotherapy and guide the development of new treatment options.

## 2 Materials and methods

### 2.1 Patients

Tumor tissue was collected from 399 patients who underwent surgery for stage I–IV NSCLC at Oslo University Hospital 2006–2016. Adjuvant treatment with a platinum‐doublet was given according to national guidelines. Information related to clinical outcome has been collected. Patient characteristics are shown in Table 1.

**Table 1:** Patient characteristics

	AD (*n* = 201)	SCC (*n* = 198)
Stage		
I	57.7% (*n* = 116)	55.6% (*n* = 110)
II	21.9% (*n* = 44)	30.3% (*n* = 60)
III	19.4% (*n* = 39)	13.6% (*n* = 27)
IV	1% (2)	0.5% (*n* = 1)
Age at surgery (years)		
Mean	66.3	67.2
Median	66.4	67.3
Range	39.2–87.0	43.2–82.4
Smoking status		
Current smoker	32.8% (66)	49.0% (97)
Ex‐smoker	54.7% (110)	50.0% (99)
Never smoker	12.4% (25)	1% (2)
Packyears		
Mean	27.0	40.4
Range	0–76	0–145 (5 missing)
Sex		
Female	54.7% (110)	33.3% (66)
Male	45.3% (91)	66.7% (132)
Recurrence	48.3% (97)	36.9% (73)
At time of recurrence		
Local recurrence	26.8% (26)	35.6% (26)
Metastases	73.2% (71)	64.4% (47)
Follow‐up time (months)		
Median	89.2	65.8
Range	18.0–128.6	16.6–136.9
OS	47.0% (1 lost to follow‐up)	51.0%

The study was approved by the regional ethics committee (REC South East), and informed written consent was obtained from all patients before surgery. The study was performed in agreement with the standards established by the Declaration of Helsinki.

### 2.2 Gene expression assessment

All samples were fresh‐frozen and stored at −80°. Gene expression was assessed using Agilent microarray on samples from AD (*n* = 184) and SCC (*n* = 183) separately. RNA sequencing (RNA seq) was performed on the remaining 32 samples, of which 17 were AD and 15 were SCC. In addition, 47 samples were collected from normal lung. Nineteen samples were derived during surgery for SCC and 28 samples during surgery for AD. These were analyzed, using Agilent microarray, together with their paired tumor sample.

#### 2.2.1 RNA sequencing

RNA was isolated with QIAcube. Concentration was measured spectrophotometrically with NanoDrop, and RNA quality was controlled using 2100 Bioanalyzer microfluidic gel electrophoresis system (Agilent, Santa Clara, CA, USA). Two samples were excluded due to low RNA quality. The remaining 32 samples all had a RIN value above 6 (mean: 9.2) and were accepted for further analysis.

An RNA seq library was prepared using TruSeq stranded mRNA sample prep kit (Illumina, San Diego, CA, USA). In short, RNA was mRNA enriched using oligodT bead system (Illumina). Double‐stranded cDNA was synthesized, end repaired, 3′adenylated, and Illumina sequencing adaptors were ligated onto the fragments ends (Agencourt AMPure XP; Beckman Coulter, Brea, CA, USA). The libraries were pre‐amplified with PCR (Agencourt AMPure XP) and then validated and quality inspected on the 2100 Bioanalyzer and quantified using the Qubit fluorometer (Life Technologies, Waltham, MA, USA).

Sequencing was performed on NextSeq500 instrument (Illumina). Raw reads were evaluated using fastqc (v0.11.3; Babraham institute, Cambridge, UK). trimmomatic (v0.35; Bolger *et al*., 2014) was used to remove adapter content and to trim reads with very low quality. The reads were then mapped to a reference genome using tophat (v2.1.0; Kim *et al*., 2013) and assembled into genes using htseq (v0.6.1; Anders *et al*., 2015). Fragments per kilobase per million read (FPKM) values were calculated.

#### 2.2.2 Microarray data

RNA was extracted and analyzed as previously described (Bjaanaes *et al*., 2016). In short, RNA isolation was done using standard TRIZOL methods and quantity and quality was controlled using the NanoDrop ND‐1000 spectrometer and the 2100 Bioanalyzer. Gene expression was assessed using gene expression microarrays from Agilent technologies (SurePrint G3 human GE 8 × 60 K and SurePrint G3 human GE v3 8 × 60 K for the AD and SCC respectively). The raw data were processed with the agilent's feature extraction Software with default parameters (Agilent feature extraction version 10.7.3.1). Probes were collapsed by median, samples were quantile normalized, and the data were log2 transformed. Since the samples from AD and SCC were analyzed using different versions of the platform, they were normalized separately.

### 2.3 Immunohistochemistry

Information on tumor histology and stage was obtained from the patient records.

In 335 samples, PD‐L1 expression was evaluated by immunohistochemistry (IHC). All tumors were formalin fixed and paraffin embedded. Representative tumor tissue was selected from Hematoxylin–Eosin stained slides. In the first 29 samples, 3‐μm thin sections were treated in PT‐link with FLEX Target Retrieval Solution, High pH (pH 9.0). En Vision FLEX Peroxidase‐Blocking Reagent (0.03% H_2_O_2_) was used for inhibition of endogenous peroxidase. The slides were then stained with anti‐PD‐L1 (405.9A11) mouse monoclonal antibody (Cell Signaling Technology, Danvers, MA, USA, clone 1543a, Lot 1 August 2018). Flex+ (Dako, Agilent) was used as detection system. In the remaining samples, PD‐L1 expression was assessed in tissue micro array (TMA) blocks. At least two 1 mm punch biopsies were stained with PD‐L1 antibody 22C3. Proportion of PD‐L1 positive tumor cells was manually evaluated by an experienced pathologist.

### 2.4 *TP53*,* EGFR,* and *KRAS* mutation assessment

The *TP53* mutation in exon 2–11 was analyzed by Sanger sequencing using the AB 3730 DNA Analyzer (Applied Biosystems, Waltham, MA, USA) after standard protocol. The wobble‐enhanced ARMS method was used for detecting the seven most commonly reported *KRAS* mutations. *EGFR* mutations were analyzed using the TheraScreen EGFR mutation kit (DxS, Manchester, UK) designed to detect 28 specific mutations in the *EGFR* gene. The results are previously published and methods more thoroughly described in the respective publications (Halvorsen *et al*., 2016; Hamfjord *et al*., 2011; Helland *et al*., 2011).

### 2.5 Statistics

Adenocarcinomas were classified as TRU (formerly bronchioid), PP (formerly magnoid) or PI (formerly squamoid), and SCC as basal, secretory, primitive, or classical using the previously published centroid classifiers for AD (Wilkerson *et al*., 2012) and SCC (Wilkerson *et al*., 2010), respectively. Microarray data were quantile normalized, log2 transformed, and median centered. For the RNA seq dataset, FPKM values were upper quantile normalized, log2 transformed, median centered, and the samples were split in two groups according to histology prior to classification. Subtypes were assigned using Pearson correlation. Four of the microarray AD had negative correlation for all subtypes and were not assigned a subtype.

xCell (Aran *et al*., 2017) was used to estimate the presence of different immune cells in tumor and to calculate an immune score. The RNA seq dataset and the two microarray datasets were analyzed separately. The microarray datasets were quantile normalized, but the probes were not collapsed as this is done by default, using averages, in xCell. For the RNA seq dataset, FPKM values were used.

For calculating cytolytic score, proliferation score and to compare expression of *CD274,* the three datasets were merged and quantile normalized together. Cytolytic score was calculated as the geometric mean of *GZMA* and *PRF1* (Rooney *et al*., 2015). Tumor proliferation was calculated using the cell cycle progression (CCP) score, which uses the average of 31 cell cycle genes (Wistuba *et al*., 2013). As a quality control cytolytic score, CCP score and expression of *CD274* before and after merging and quantile normalizing were plotted in the three datasets separately, revealing good visual correlation and a Pearson correlation coefficient close to 1.

Heatmaps were created using r software developed by one of the authors (OCL). Immune cell type estimates were median centered before creating heatmaps. Hierarchical clustering was based on Euclidean distance with comple linkage.

All comparisons of estimated immune cell types, immune score, cytolytic score, and proliferation score between groups were made using Wilcoxon rank sum test, if nothing else is declared. Comparisons between more than two groups were made using the Kruskal–Wallis test.

The impact of different factors on PFS, defined as time to relapse after surgery, was analyzed using Cox proportional regression analysis. Patients who died of other causes than lung cancer during follow‐up were censored at time of death. IHC PD‐L1 expression, *CD274* gene expression, cytolytic score, CCP score, immune score, *CD274* gene expression/immune score, and all estimates of immune cell types were divided by their standard deviation (SD) to achieve comparable estimates of hazard ratio (HR).

All statistical analyses were performed using r version 3.4.3 (http://www.r-project.org).

## 3 Results

### 3.1 Immune microenvironment in histological subgroups

**Figure 1:**
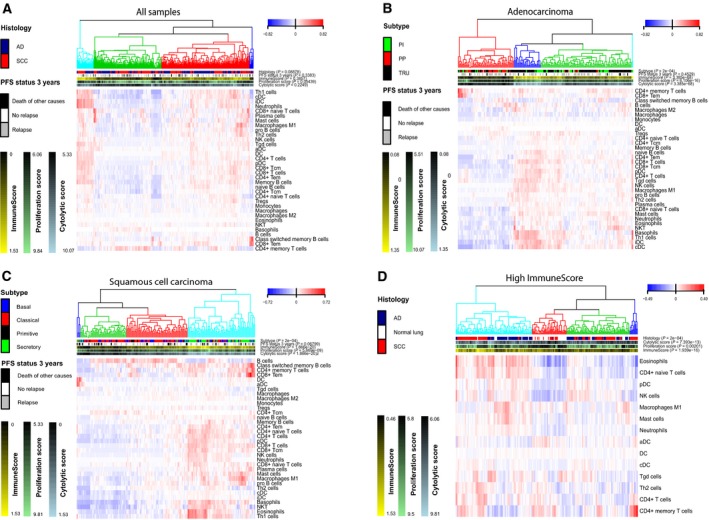
Heatmaps based on estimates of 34 immune cell types in (A) all samples, (B) AD, and (C) SCC. In the last heatmap (D), only immune cell types found to be significantly different between AD and SCC with immune score above median are used. Tumor samples with xCell immune score above median, and all samples from normal lung, are included in the heatmap. CD8+ Tem, CD8+ effector memory T cells; CD4+ Tcm, CD4+ central memory T cells; NKT, natural killer T cells.

xCell was used to calculate an immune score and to get an estimate of different cell types in the tumor microenvironment. Of 64 cell types, 34 were immune cells. These were median centered and used to cluster the samples (Fig. 1A). We could see that samples with high immune score clustered together and that both AD and SCC were found in the most immune cell‐rich clusters. xCell estimates of immune cells were also used to cluster AD and SCC separately (Fig. 1B,C).

Immune cell type estimates were compared between AD and SCC with immune score above median (median immune score = 0.6847, AD *n* = 92, SCC *n* = 107) using Wilcoxon rank sum test with Bonferroni correction for multiple testing. We found significantly higher estimates of eosinophils, macrophages M1, neutrophils, CD4+ naive T cells, and plasmacytoid DC (pDC) in SCC with high immune score and more gamma delta T cells (Tgd cells), conventional DC (cDC), mast cells, NK cells, CD4+ T cells, DC, activated DC (aDC), type 2 T‐helper cells (Th2 cells), and CD4 memory T cells in AD with high immune score. In order to evaluate the immune cell distribution in histological subgroups with high immune score compared to normal lung, cell types with significantly different estimates in AD and SCC were plotted in a heatmap together with 47 samples from normal lung (Fig. 1D). We could then see that 31/47 samples from normal lung clustered together. One AD and 15 SCC clustered with normal lung.

**Figure 2:**
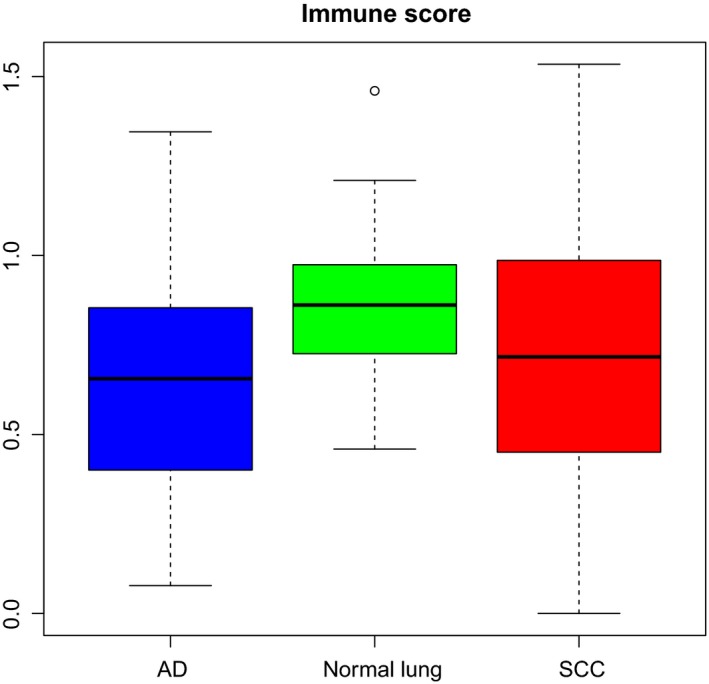
Box plot showing immune score in AD, SCC, and normal lung.

The xCell immune score, cytolytic score, and CCP proliferation score were compared between AD and SCC, as shown in Table 2 and Fig. 2. Correlation tests between scores revealed that immune score and cytolytic score were positively correlated in both AD and SCC [SCC: Pearson correlation coefficient (*r*) = 0.63 and *P* < 0.001 AD: *r* = 0.58 and *P* < 0.001]. Proliferation score was negatively correlated to immune score (*r* = −0.574 and *P* < 0.001) and cytolytic score (*r* = −0.33 and *P* < 0.001) in SCC while in AD we found no significant correlation.

**Table 2:** xCell immune score, CCP proliferation score, and cytolytic score in histological subgroups

	AD	SCC
xCell immune score		
Mean (*T*‐test *P*: 0.016)	0.65	0.73
Median (range)	0.66 (0.078–1.35)	0.72 (0.00–1.53)
CCP proliferation score		
Mean (*T*‐test *P*: 3.488e‐12)	7.77	8.40
Median (range)	7.75 (6.06–9.63)	8.71 (6.11–9.84)
Cytolytic score		
Mean (*T*‐test *P*: 0.013)	7.42	7.22
Median (range)	7.44 (5.51–10.07)	7.24 (5.33–9.81)

Estimates of immune cells were also compared between all AD and SCC, and results can be found in Appendix S1.

### 3.2 Gene expression subtypes

Adenocarcinomas and SCC were investigated separately to see whether clustering based on immune cell type estimates would separate the samples according to their expression subtype (Fig. 1B–C). We found that 41 out of 48 secretory SCC clustered together in the cluster with the highest immune score. No obvious pattern was seen for the other subtypes. In AD, 29 out of 36 PP samples clustered together in the cluster with the lowest immune score. Also, 35 out of 44 PI samples and 92 out of 117 TRU samples were found in the three most immune cell‐rich clusters. Cluster number two had high immune score, was dominated by the TRU subtype (24 out of 31 samples), and did not contain any PP AD. We also performed clustering of all samples, including normal lung, and found the AD TRU subtype and the SCC secretory subtype to be most similar to normal lung (Fig. S1A–C). Notably, normal samples did not cluster with their paired tumor sample (Fig. S1D).

Estimates of immune cells in expression subtypes were compared to normal lung using Wilcoxon rank sum test with Bonferroni correction for multiple testing (Appendix S1). In SCC, we found that the secretory expression subtype had high estimates of most immune cells. Only mast cells, cDC and NK cells were significantly higher in normal lung compared to in the secretory subtype. In the primitive, basal, and classical subtypes, some cell types were elevated compared to normal lung, but there was no convincing pattern of adaptive cell types being more prominent in tumor samples than in normal lung (Fig. S2).

In AD, the same pattern was seen for almost all immune cells with low estimates in the PP subtype and high in the TRU and PI subtypes. When comparing TRU and PI to normal lung, the same cell types were elevated in tumor samples. These included aDC, CD8+ central memory T cells (CD8+ Tcm) cells, M1 macrophages, B cells, plasma cells, Th2 cells, memory B cells, naïve B cells, pro B cells, type 1 T‐helper cells (Th1 cells), and Tgd cells. In the TRU subtype, there were in addition higher estimates of class‐switched memory B cells.

Proliferation score, cytolytic score, and immune score differed in the expression subtypes as shown in Table 3.

**Table 3:** xCell immune score, CCP proliferation score, cytolytic score, *CD274* gene expression, and *CD274* gene expression/xCell immune score in expression subtypes and normal lung. IHC PD‐L1 expression and *EGFR*,* TP53,* and *KRAS* mutation status in expression subtypes. *CD274* gene expression/xCell immune score was not possible to calculate in one SCC sample with the classical expression subtype due to zero immune score. ‘*P*‐value' refers to *P*‐value when scores/estimates in the expression subtypes were compared to normal lung using Wilcoxon rank sum test

Expression subtype (*n*)	Basal (56)	Classical (85)	Primitive (9)	Secretory (48)	PI (44)	PP (36)	TRU (117)	Normal lung (47)
xCell immune score								
Mean	0.73	0.55	0.53	1.07	0.75	0.32	0.72	0.88
Median	0.76	0.48	0.58	1.13	0.72	0.32	0.73	0.86
*P*‐value	0.0073	1.7e‐09*	0.00037*	1.0e‐05*	0.013	4.5e‐20*	0.00047*	
CCP proliferation score								
Mean	8.45	9.01	8.87	7.20	8.39	8.43	7.33	6.22
Median	8.55	9.04	9.15	6.92	8.54	8.49	7.32	6.18
*P*‐value	3.0e‐18*	2.3e‐21*	2.6e‐10*	2.5e‐17*	1.0e‐26*	4.9e‐24*	9.4e‐21*	
Cytolytic score								
Mean	7.19	6.95	7.00	7.79	7.80	6.99	7.42	7.61
Median	7.21	6.95	6.93	7.77	7.75	6.87	7.46	7.67
*P*‐value	0.00071*	7.5e‐08*	0.011	0.11	0.33	2.8e‐05*	0.087	
*CD274* gene expression								
Mean	6.59	6.92	6.58	6.73	7.43	6.25	6.40	6.63
Median	6.44	6.81	6.25	6.77	7.35	6.02	6.28	6.63
*P*‐value	0.24	0.19	0.12	0.59	1.3e‐05*	0.0041	0.016	
*CD274* gene expression/xCell immune score								
Mean	12.57	41.90	39.29	7.77	12.09	27.33	10.99	7.89
Median	8.28	14.67	10.52	6.08	10.44	18.71	8.92	7.81
*P*‐value	0.019	2.4e‐11*	0.00016*	1.6e‐06*	7.6e‐05*	4.5e‐20*	0.0024	
IHC PD‐L1 expression								
*N*	38	71	7	31	39	34	111	
Mean	8.39	15.89	12.14	13.68	30.0	8.94	8.05	
PDL1 positive (%)	10 (26.3)	29 (40.8)	1 (14.3)	8 (25.8)	20 (51.3)	9 (26.5)	25 (22.5)	
*TP53* mutation status								
*N*	26	48	5	26	40	29	110	
Nonsilent mutation (%)	16 (62)	37 (77)	5 (100)	13 (50)	18 (45)	13 (45)	42 (38)	
*EGFR* mutation								
*N*					43	36	116	
Mutation (%)					0 (0)	1 (2.8)	26 (22.4)	
*KRAS* mutation								
*N*	21	38	4	21	37	26	105	
Mutation (%)	0 (0)	2 (5.3)	1 (25)	1 (5)	16 (43.2)	5 (19.2)	43 (41.0)	

* Indicates *P*‐values that stayed significant after Bonferroni correction for multiple testing including all tests in the table.

### 3.3 TP53 mutation status

*TP53* mutation status was available for 105 SCC and 183 AD, and of these, 71 (67.6%) and 75 (41.0%) had nonsilent mutations, respectively. For proportion of samples mutated in expression subtypes, see Table 3. We investigated differences in immune cell distribution in samples with or without a *TP53* mutation. After Bonferroni correction for multiple testing, samples with a nonsilent mutation had higher estimates of neutrophils, Tgd cells, and Th1 cells and lower estimates of CD4+ effector memory T cells (CD4+ Tem), DC, immature dendritic cells (iDC), Tregs, and cDC. The same pattern was seen when AD and SCC were tested separately but only Th1 cells, Tgd cells, and CD4+ Tem in SCC and Th1 cells in AD remained significant after correction for multiple testing. We also looked for differences in immune score, cytolytic score, and proliferation and found higher proliferation in those having a *TP53* mutation in AD, SCC, and all samples tested together. Immune score was elevated in nonmutated samples in SCC.

### 3.4 PD‐L1

Programmed death‐ligand 1 expression by IHC was analyzed for 335 samples (188 AD and 147 SCC), and of these, 103 (55 AD and 48 SCC) were PD‐L1 positive (> 1% positive tumor cells) and 41 (23 AD and 18 SCC) had more than 50% PD‐L1‐positive tumor cells. As expected, IHC PD‐L1 expression correlated with *CD274* gene expression (*r* = 0.58 and *P* < 2.2e‐16).

Correlation analyses were performed to investigate what characteristics of the immune microenvironment were important for the expression of PD‐L1. Results for IHC PD‐L1 expression and *CD274* gene expression were highly overlapping, but correlation was stronger when we used *CD274* gene expression. This is probably reflecting that more samples were available for analyses on gene expression and that many samples were PD‐L1 negative when assessed by IHC. The strongest positive correlation with IHC PD‐L1 expression and *CD274* gene expression was seen with macrophages M1, cytolytic score, NK cells, and CD8+ Tcm cells, and the same pattern was seen when AD and SCC were tested separately.

*CD274* gene expression was not found to be significantly different in histological subgroups when tested using a two‐sided *t*‐test. In Table 3, *CD274* gene expression, immune score, and *CD274* gene expression per immune score in expression subtypes are shown and compared to normal lung.

### 3.5 Progression‐free survival analysis

Median follow‐up time was 71.6 months (85.6 months for AD and 58.5 months for SCC) calculated as median observation time for patients with no relapse when data were collected. Twenty‐eight patients with AD and 39 patients with SCC died from other causes during follow‐up. We found no difference in PFS in SCC compared to AD [HR: 0.80 95% confidence interval (CI): 0.59–1.09, *P* = 0.161]. There was not seen any significant difference in PFS reflecting age at surgery, smoking status, packyears, sex, or stage in SCC. In AD, we found more advanced stage to be associated with poor prognosis.

Expression subtype did not predict PFS in SCC. In AD, patients with the expression subtype TRU had a better prognosis (HR: 0.50, *P* = 0.000853) versus non‐TRU and this stayed significant after adjusting for stage (HR: 0.59, *P* = 0.01222). *TP53* mutation status had no significant impact on PFS when tested in the whole cohort and in histological subgroups and expression subtypes (not possible to calculate in the SCC primitive subtype where only five samples were available and all had a *TP53* mutation).

Immunohistochemistry PD‐L1 expression and *CD274* gene expression were investigated as prognostic markers, adjusting for stage. We did not find any difference in PFS according to *CD274* gene expression or IHC PD‐L1 expression levels in histological subgroups. In expression subtypes, increasing *CD274* gene expression was associated with better prognosis in the secretory subtype and with poor prognosis in the classical subtype. In the classical subtype, results were also significant for IHC PD‐L1 expression.

Immune score, cytolytic score, and proliferation score did not affect PFS in SCC or any of the SCC expression subtypes. In AD, increasing immune score and decreasing proliferation were associated with better PFS while cytolytic score only came out significant in the PP expression subtype, all adjusted for stage.

Results for PFS analyses on clinical and molecular characteristics in histological subgroups and expression subtypes, as described above, can be found in Tables 4, [Link], [Link].

**Table 4:** PFS in histological subgroups. PFS assessed by Cox proportional regression analysis

	Adenocarcinoma		SCC	
	HR (95% CI)	*P*‐value	HR (95% CI)	*P*‐value
Stage I–IV	1.57 (1.26–1.97)	6.43e‐05*	1.26 (0.92–1.71)	0.15
Smoking status				
Current smoker	1		1	
Previous smoker	1.22 (0.78–1.92)	0.386	1.22 (0.77–1.94)	0.405
Never smoker	1.19 (0.62–2.30)	0.604	4.21 (0.57–31.37)	0.160
Age at surgery	1.01 (0.98–1.03)	0.51	1.00 (0.97–1.03)	0.799
Packyears	1.00 (0.98–1.01)	0.530	1.01 (1.00–1.02)	0.255
Sex				
Male	1		1	0.557
Female	0.72 (0.48–1.09)	0.119	1.16 (0.71–1.9)	
Expression subtype	PI: 1		Basal: 1	
	PP: 1.40 (0.79–2.46)	0.250	Classical: 0.94 (0.54–1.63)	0.821
	TRU: 0.58 (0.36–0.95)	0.0287*	Primitive: 1.51 (0.57–4.01)	0.407
			Secretory: 0.82 (0.42–1.59)	0.555
TP53 mutation^a^				
Wild‐type or silent mutation	1		1	0.412
Nonsilent mutation	1.19 (0.78–1.81)	0.430	1.32 (0.68–2.59)	
KRAS mutation^a^				
Wild‐type	1		1	0.684
Mutation	1.39 (0.91–2.15)	0.130	1.35 (0.32–5.72)	
EGFR mutation^a^				
Wild‐type	1		_	_
Mutation	1.06 (0.62–1.82)	0.82		
Immune score^a^	0.78 (0.63–0.96)	0.0196*	0.99 (0.78–1.25)	0.923
Cytolytic score^a^	0.85 (0.69–1.05)	0.122	0.84 (0.67–1.07)	0.164
Proliferation score^a^	1.27 (1.03–1.57)	0.0231*	1.09 (0.86–1.39)	0.458
*CD274* gene expression^a^	1.01 (0.82–1.24)	0.956	1.08 (0.85–1.37)	0.513
IHC PD‐L1^a^	1.06 (0.87–1.29)	0.559	1.13 (0.89–1.44)	0.302

a Adjusted for stage.

* *P *<* *0.05.

Progression‐free survival was also compared between the main clusters formed in the heatmaps described in section 3.3. We did not find any significant difference in PFS between the four main clusters in Fig. 1A. In Fig. 1B,C, the samples were divided according to histological subgroup and clustered. There was no significant difference in PFS between the main clusters in SCC. In AD, the cluster with the lowest immune score had shorter PFS (HR: 1.75, *P* = 0.007; adjusted for stage: HR: 1.52, *P* = 0.0481) and the cluster with the highest immune score close to significant longer PFS (HR: 0.53, *P* = 0.061; adjusted for stage: HR: 0.63, *P* = 0.176). In Fig. 1D, showing samples with immune score above median, there was no difference in PFS between the clusters when AD and SCC were tested separately, adjusting for stage.

**Figure 3:**
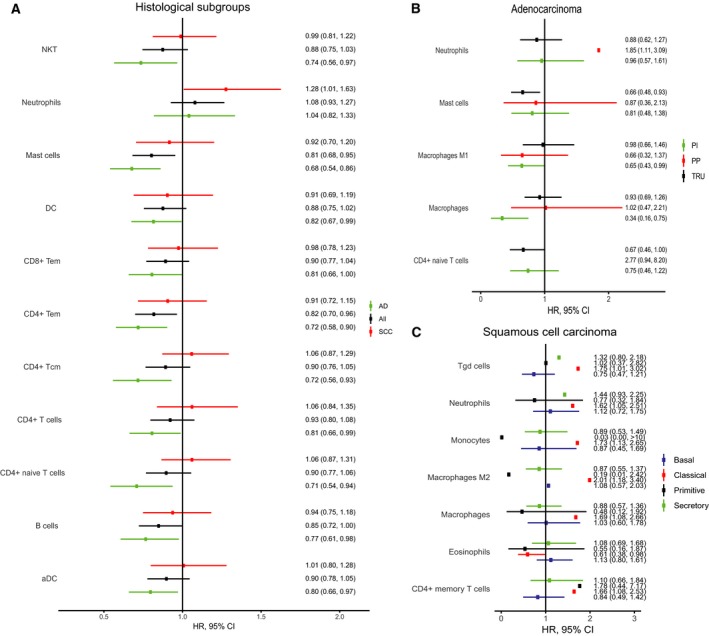
HR and 95% CIs for PFS in histological subgroups and expression subtypes per one SD change in immune cell type estimates, adjusted for stage. CD8+ Tem, CD8+ effector memory T cells; CD4+ Tcm, CD4+ central memory T cells; NKT, natural killer T cells.

The 34 immune cell types estimated by xCell were tested for relation to PFS in histological subgroups and expression subtypes, adjusting for stage (Fig. 3A–C). To further investigate the impact of the immune microenvironment on prognosis, we compared samples from patients with and without relapse, local recurrence, and metastases (see Appendix S1). In those with local recurrence, we found higher proliferation, CD8+ naïve T cells, and Th1 cells and lower immune score, CD4+ Tem, CD4+ naïve T cells, eosinophils, iDC, B cells, M2 macrophages, Tregs, and class‐switched memory B cells compared to those without relapse. In AD, a similar pattern was found, while in SCC this was not seen. Results indicate that the immune activity in tumor is of greater importance in preventing local recurrence than metastatic disease, though some patients might have had not yet detected metastases at time of surgery, blurring the results.

## 4 Discussion

Patients with NSCLC eligible for curative surgery still have a bleak prognosis. In our material, with 399 samples from previously untreated lung AD and SCC, we found clinical and molecular characteristics predictive of risk of recurrence. In AD, we found decreasing stage, decreasing proliferation, increasing immune score, and the TRU expression subtype to be associated with better prognosis. Stage is known to be the most important prognostic factor in both AD and SCC, but in our material this was not confirmed in SCC. The reason for this might be that there were few patients with stage III in the SCC group.

When samples were clustered based on estimates of immune cells, we could see that both histological subgroups were represented in all four clusters derived. We also found xCell immune score to be similarly distributed in AD and SCC. This pointed toward that the degree of immunological activity mainly varied within, and not between, histological subgroups. When AD and SCC were clustered separately, we found poor PFS in the AD cluster with the lowest immune score, consistent with that increasing immune score had been found to be a good prognostic factor in AD. Since this was not the case in SCC, we wanted to see whether there were differences between the immune microenvironment in AD and SCC with high immune score, hypothesizing that it would be more dominated by an adaptive immune response in AD. We found this to be correct. When comparing samples from normal lung to tumor samples with high immune score, we also saw that more SCCs than ADs had an immune cell distribution similar to normal lung.

In expression subtypes, earlier findings of more immunological activity in the AD TRU and PI expression subtypes and the SCC secretory expression subtype were confirmed (Faruki *et al*., 2017). In AD, all expression subtypes showed higher estimates of the three T effector cells Th1 cells, Th2 cells, and Tgd cells compared to normal lung. In addition, they had more plasma cells. This indicates that there is an adaptive immune response in AD regardless of expression subtype, though in the PP subtype the overall immune activity is low. In SCC, on the other hand, the immune cell‐rich secretory subtype shared many immune characteristics with normal lung, possibly explaining why increasing immune score and immune cell estimates were not associated with better prognosis in SCC. The classical subtype had high proliferation and PD‐L1 expression but low immune score and cytolytic score indicating aggressive disease with an inhibited antitumor immune response. This was supported by that increasing PD‐L1 expression and increasing estimates of some immune cell types including the immunosuppressive macrophages M2 were associated with poor prognosis in the classical subtype.

The lung is an organ that is constantly exposed to the outer world and is, not surprisingly, rich in immune cells that form an effective first defense against microbes and foreign particles. We therefore found it valuable to have samples from normal lung available, when trying to identify tumor‐specific immune properties. One could argue that samples derived from normal lung during surgery for NSCLC are not optimal as controls since most lung cancer patients are smokers and this could affect the immune microenvironment. However, the immune response seen in smokers compared to in nonsmokers is different from what we expect to find in a tumor (Grumelli *et al*., 2004; Shaykhiev *et al*., 2009), and when samples from normal lung were clustered together with tumor samples based on immune cell estimates, these did not cluster with their paired tumor sample.

*TP53* is a well‐known tumor suppressor gene, commonly found mutated in NSCLC. As a prognostic factor, *TP53* mutations predict worse OS in most studies (Deben *et al*., 2016; Kosaka *et al*., 2009). In our material, we did not find any significant impact of *TP53* mutation status on PFS. Since *TP53* is an important DNA repair gene, we expect those having a *TP53* mutation to have more mutations, resulting in more antigens and a stronger immune response. We did not find higher immune score in tumors with a *TP53* mutation but higher proliferation, indicating more aggressive disease. There are increasing evidence that *TP53* functions as an immune modulator affecting both cytokine production, immune cell function, and expression of immune checkpoints (Munoz‐Fontela *et al*., 2016). We found lower estimates of DC, CD4+ Tem and Tregs, and higher estimates of neutrophils, Tgd cells, and Th1 cells in samples with a nonsilent *TP53* mutation. Lacking *TP53*‐mediated immune activation, and especially insufficient activation of DC which are crucial for an effective antitumor immune response, might explain why we did not find higher immune score in *TP53*‐mutated samples.

Immune cell content can be estimated from gene expression using gene set enrichment analysis or deconvolution methods. None of these methods are perfect. xCell (Aran *et al*., 2017) uses multiple gene signatures to estimate content of immune and stromal cells, excluding genes known to be overexpressed in cancer. Still, untypical gene expression patterns in tumor cells are a source of inaccurateness when studying tumor samples, and this can explain why we sometimes get small estimates of cell types not likely present in the tumor microenvironment. Localization of the different immune cells in tumor has also been associated with patient outcome (Badalamenti *et al*., 2018). In our study, we did not have whole sections from the tumors, which hindered us in such investigations.

Lately, there has been great interest in identifying tumors with high cytolytic activity, PD‐L1 expression, and estimates of immune cells, particularly CD8+ T cells, hoping these would function as predictive biomarkers for response to immune checkpoint inhibitors. In that context, it is interesting to see that normal lung has high immune score, cytolytic score, and *CD274* gene expression. This indicates that there is a constant immunological activity in normal lung, which involves not only innate but also adaptive immune cells, and that activation of immune checkpoints is needed in normal lung to control this immune activity. In an attempt to identify tumors where inhibition of the immune response was stronger than one would expect from the overall immunological activity, *CD274* gene expression per xCell immune score was calculated. This ratio was high in the classical and primitive SCC subtypes and in the PP and PI AD subtypes. In the immune cell‐rich SCC secretory and AD TRU subtypes, CD274/immune score ratio was low. These two expression subtypes both had low proliferation, and the TRU subtype is known to have a low mutational burden (Faruki *et al*., 2017), making a strong tumor‐directed immune response in these less likely. *STK11* mutations have been associated with low PDL1 expression on tumor cells in AD (Scheel *et al*., 2016) and with lack of response to immune checkpoint inhibitors (Hellmann *et al*., 2018). Inactivating *STK11* mutations are often found in the immunologically inactive PP subtype. Neoadjuvant/adjuvant immunotherapy is most likely to influence patient outcome when tumor cells are well recognized by the immune system but activation of immune checkpoints, either by tumor cells expressing PDL1 or as a physiological part of the immune response, is the main reason why tumor is not eliminated. With this in mind, the AD PI subtype and the SCC classical and primitive subtypes might be the best candidates for successful treatment with immunotherapy.

The only predictive biomarker for response to immune checkpoint inhibitors in clinical use is IHC PDL1 expression. It can be used to select patients more likely to respond to treatment but also PDL1‐negative tumors can respond, and its predictive ability seems to be more pronounced in AD than in SCC (Borghaei *et al*., 2015; Brahmer *et al*., 2015). In our cohort, relatively few tumors were IHC PDL1 positive. There are indications that early‐stage operable tumors might have lower PDL1 expression (Lin *et al*., 2017), but could also be that PDL1‐positive tumor cells would have been found if other parts of the tumor had been used to assess this, as TMA analyses use only small cores of the tumors. The often seen heterogeneity in PDL1 expression within the same tumor (McLaughlin *et al*., 2016) is a limitation when used as a biomarker. To our knowledge, expression subtypes have not been investigated as a prognostic biomarker for response to immune checkpoint inhibitors. In this study, we have found that both the immune microenvironment and the impact it has on risk of relapse after surgery differ in histological subgroups and expression subtypes. In AD, high immune score and signs of an adaptive immune response were associated with better prognosis. It is likely that this is just as important in SCC but that we have not been able to identify which tumors are effectively targeted by the immune system and, in the context of immunotherapy, which harbor immune cells recognizing tumor cells as foreign but are unable to eliminate them. To enhance OS in surgically treated NCSLC, high‐risk patients must be identified and selected for additional treatment, at the same time as treatment‐related and non‐lung cancer‐related mortality must be minimized.

## 5 Conclusions

The tumor immune microenvironment is predictive of prognosis after surgery in lung adenocarcinoma but not in lung SCC. NSCLC gene expression subtypes differ in immune cell distribution and PD‐L1 expression, and should be investigated as a prognostic biomarker in patients treated with immune checkpoint inhibitors.

## Conflict of interest

The authors declare no conflict of interest.

## Author contributions

All authors have reviewed the data analyses, contributed to data interpretation, contributed to the writing of the report, approved the final version of the submitted report, and agree to be accountable for all aspects of the report. ÅH and ARH designed the study. ÅH and ÅÖ are responsible for clinical input. ÅÖ performed the data analyses supervised by OCL, ARH, and ÅH. All authors were involved in interpretation of the data. ÅÖ drafted the manuscript. All authors read, revised the manuscript critically, and approved the final manuscript.

## Supporting information


**Fig. S1**. Heatmaps based on estimates of 34 immune cells in (A) all samples and normal lung, (B) SCC and normal lung and (C) AD and normal lung. In the last heatmap (D) it is marked which tumor samples have a normal control and weather the normal control was taken from a patient with lung adenocarcinoma or SCC.

**Fig. S2**. Box plots showing immune cell type estimates in adenocarcinoma and SCC expression subtypes and normal lung.

**Table S1**. Progression free survival analysis in adenocarcinoma expression subtypes.

**Table S2**. Progression free survival analysis in SCC expression subtypes.

**Appendix S1**. Immune cell estimates compared using Wilcoxon rank sum test in histological subgroups, expression subtypes, according to TP53 mutation status and according to local/metastatic recurrence.
