# Supplementary material for: The immune microenvironment in non‐small cell lung cancer is predictive of prognosis after surgery
Source: Mol Oncol. 2019 Apr 10;13(5):1166–79. doi: 10.1002/1878-0261.12475 (PMC6487716; doi:10.1002/1878-0261.12475)

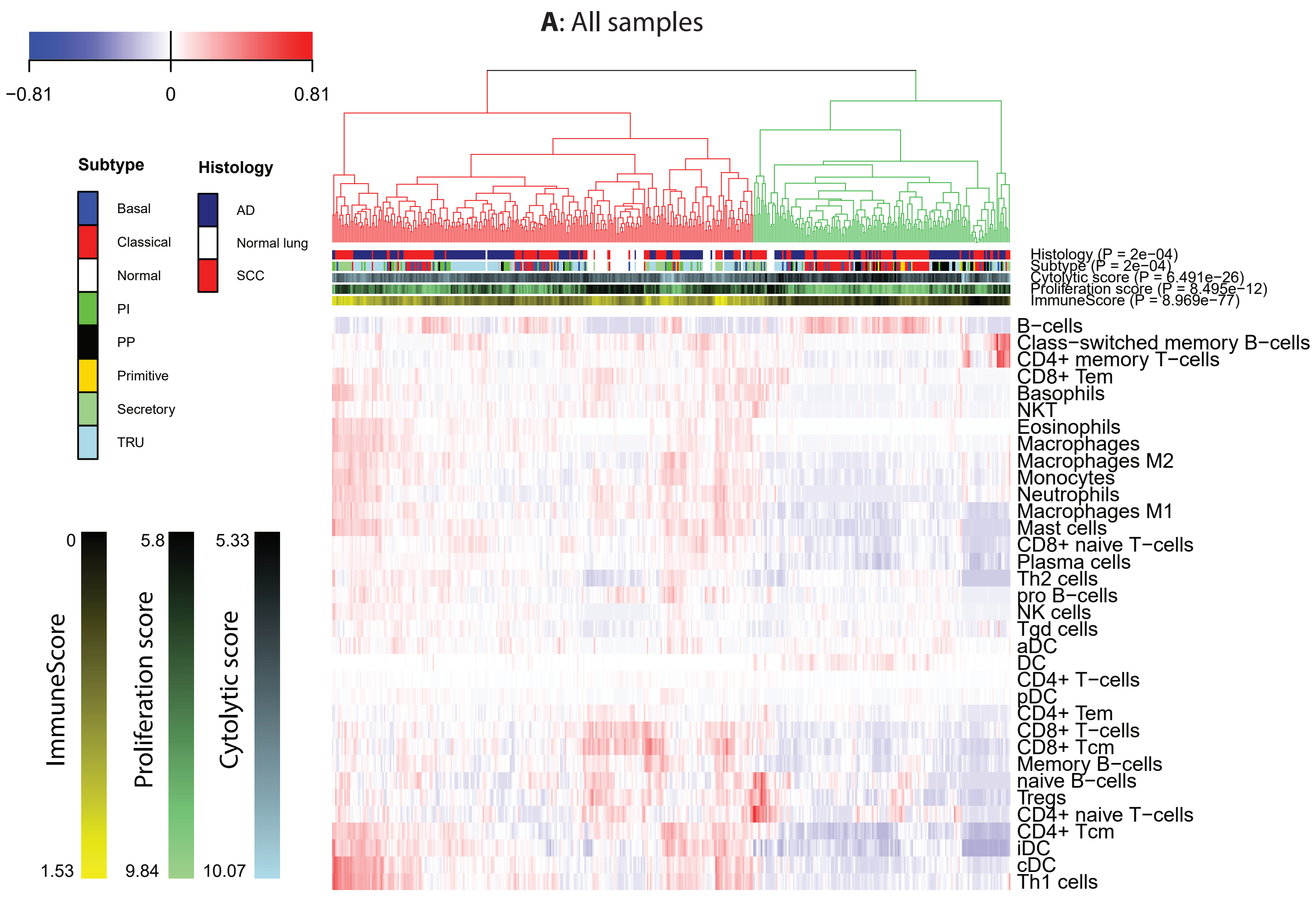
A: All samples
−0.81
0
0.81
Subtype
Basal
Classical
Normal
PI
PP
Primitive
Secretory
TRU
Histology
AD
Normal lung
SCC
ImmuneScore
0
1.53
Proliferation score
5.8
9.84
Cytolytic score
5.33
10.07
Histology (P = 2e−04)
Subtype (P = 2e−04)
Cytolytic score (P = 6.491e−26)
Proliferation score (P = 8.495e−12)
ImmuneScore (P = 8.969e−77)
B−cells
Class−switched memory B−cells
CD4+ memory T−cells
CD8+ Tem
Basophils
NKT
Eosinophils
Macrophages
Macrophages M2
Monocytes
Neutrophils
Macrophages M1
Mast cells
CD8+ naive T−cells
Plasma cells
Th2 cells
pro B−cells
NK cells
Tgd cells
aDC
DC
CD4+ T−cells
pDC
CD4+ Tem
CD8+ T−cells
CD8+ Tcm
Memory B−cells
naive B−cells
Tregs
CD4+ naive T−cells
CD4+ Tcm
iDC
cDC
Th1 cells

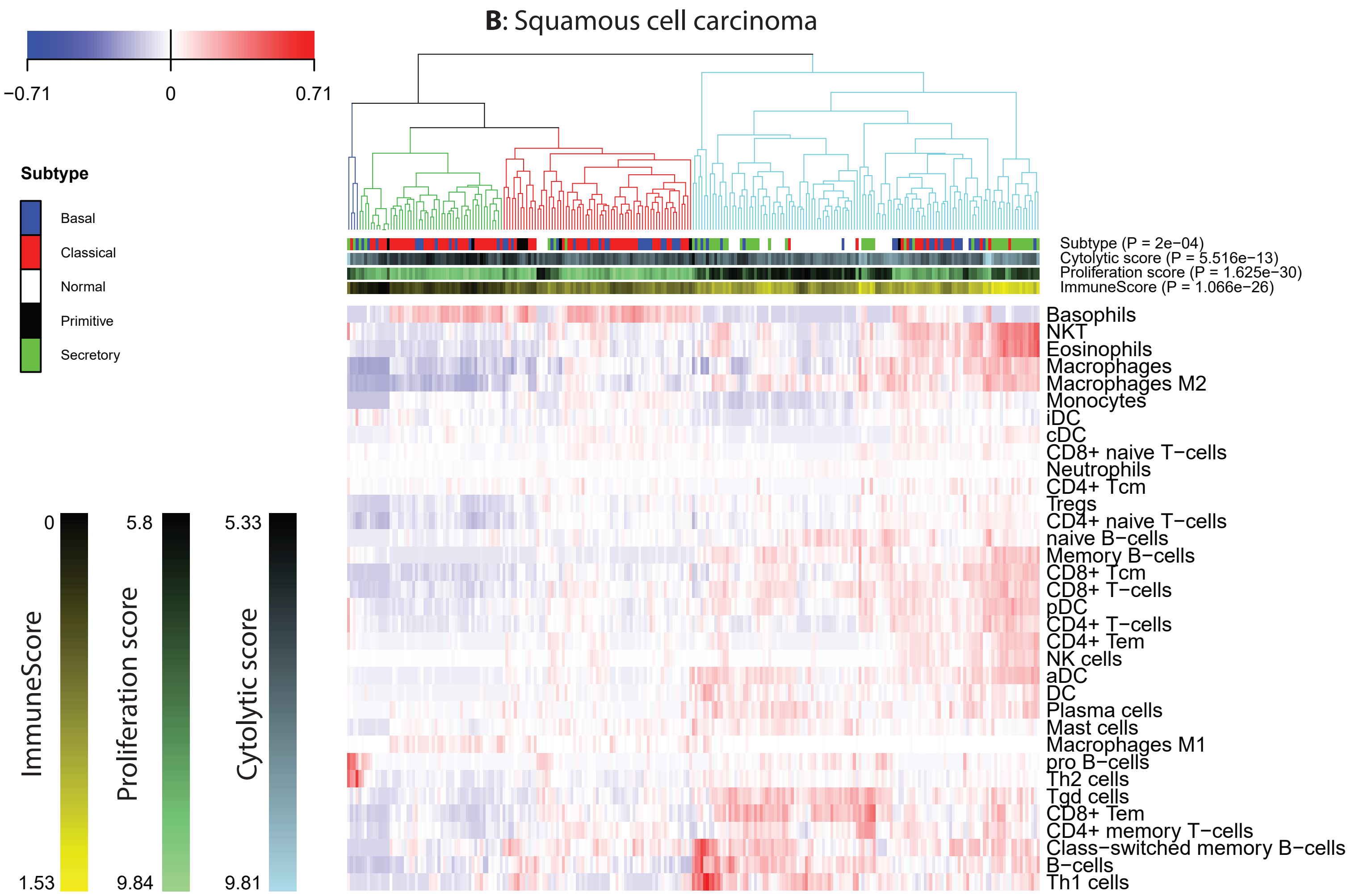
B: Squamous cell carcinoma
−0.71
0
0.71
Subtype
Basal
Classical
Normal
Primitive
Secretory
ImmuneScore
0
1.53
Proliferation score
5.8
9.84
Cytolytic score
5.33
9.81
Subtype (P = 2e−04)
Cytolytic score (P = 5.516e−13)
Proliferation score (P = 1.625e−30)
ImmuneScore (P = 1.066e−26)
Basophils
NKT
Eosinophils
Macrophages
Macrophages M2
Monocytes
iDC
cDC
CD8+ naive T−cells
Neutrophils
CD4+ Tcm
Tregs
CD4+ naive T−cells
naive B−cells
Memory B−cells
CD8+ Tcm
CD8+ T−cells
pDC
CD4+ T−cells
CD4+ Tem
NK cells
aDC
DC
Plasma cells
Mast cells
Macrophages M1
pro B−cells
Th2 cells
Tgd cells
CD8+ Tem
CD4+ memory T−cells
Class−switched memory B−cells
B−cells
Th1 cells

C: Adenocarcinoma

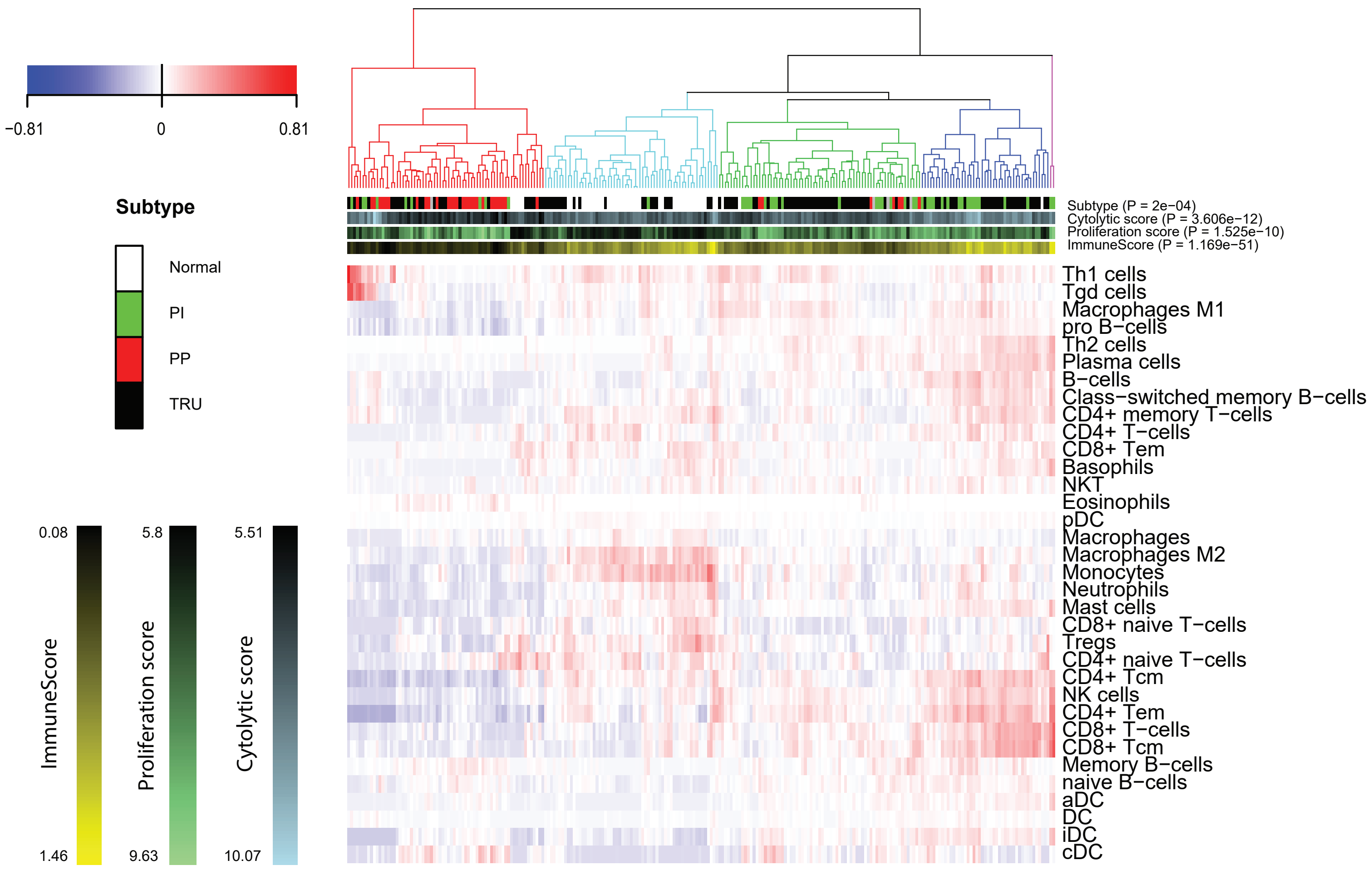

D: All samples

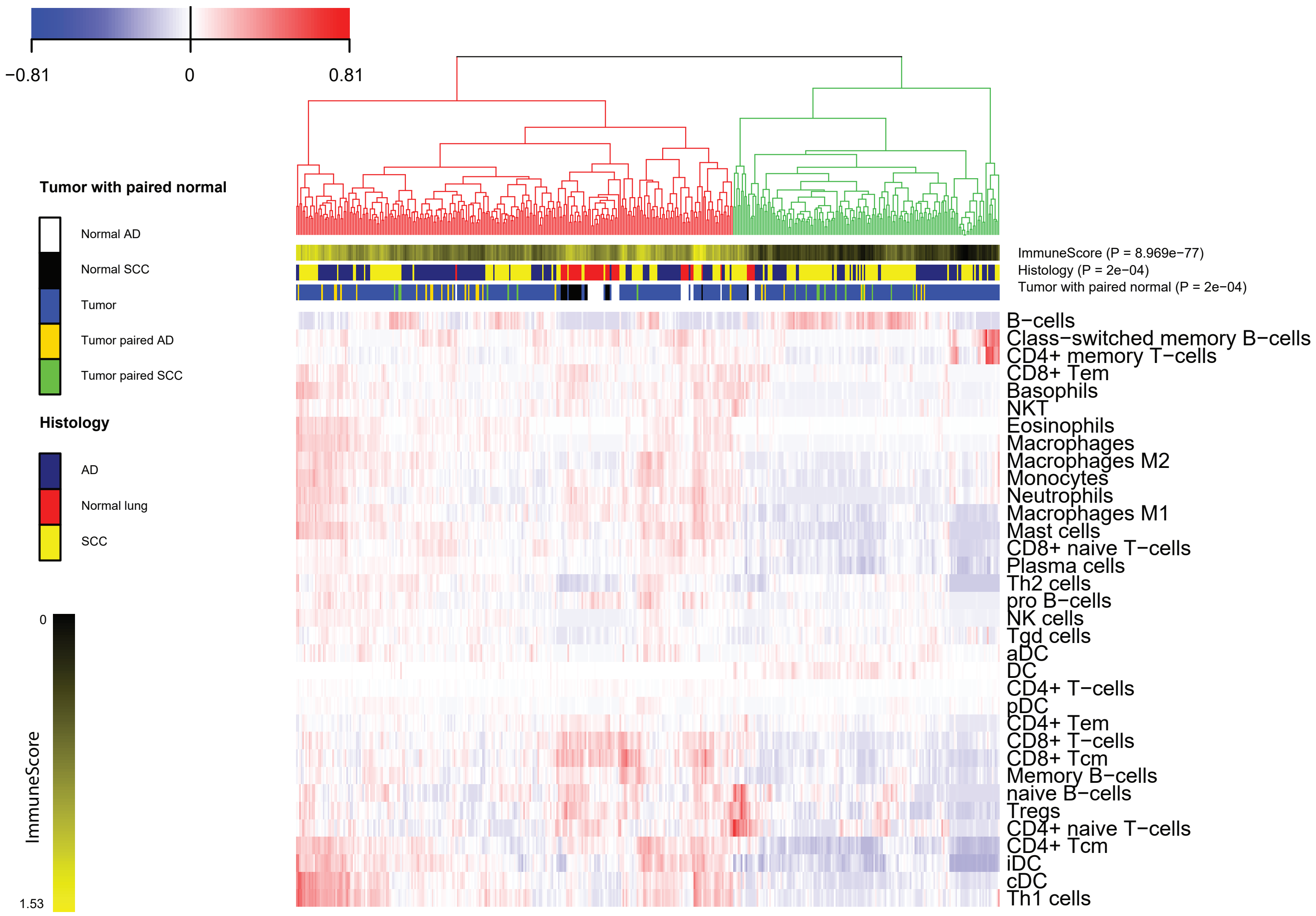

Supplement: Supplementary file 1 — Fig. S1. Heatmaps based on estimates of 34 immune cells in (A) all samples and normal lung, (B) SCC and normal lung and (C) AD and normal lung. In the last heatmap (D) it is marked which tumor samples have a normal control and weather the normal control was taken from a patient with lung adenocarcinoma or SCC. [file MOL2-13-1166-s001.pdf]
