# Supplementary figures and images for: The immune microenvironment in non‐small cell lung cancer is predictive of prognosis after surgery
Source: Mol Oncol. 2019 Apr 10;13(5):1166–79. doi: 10.1002/1878-0261.12475 (PMC6487716; doi:10.1002/1878-0261.12475)

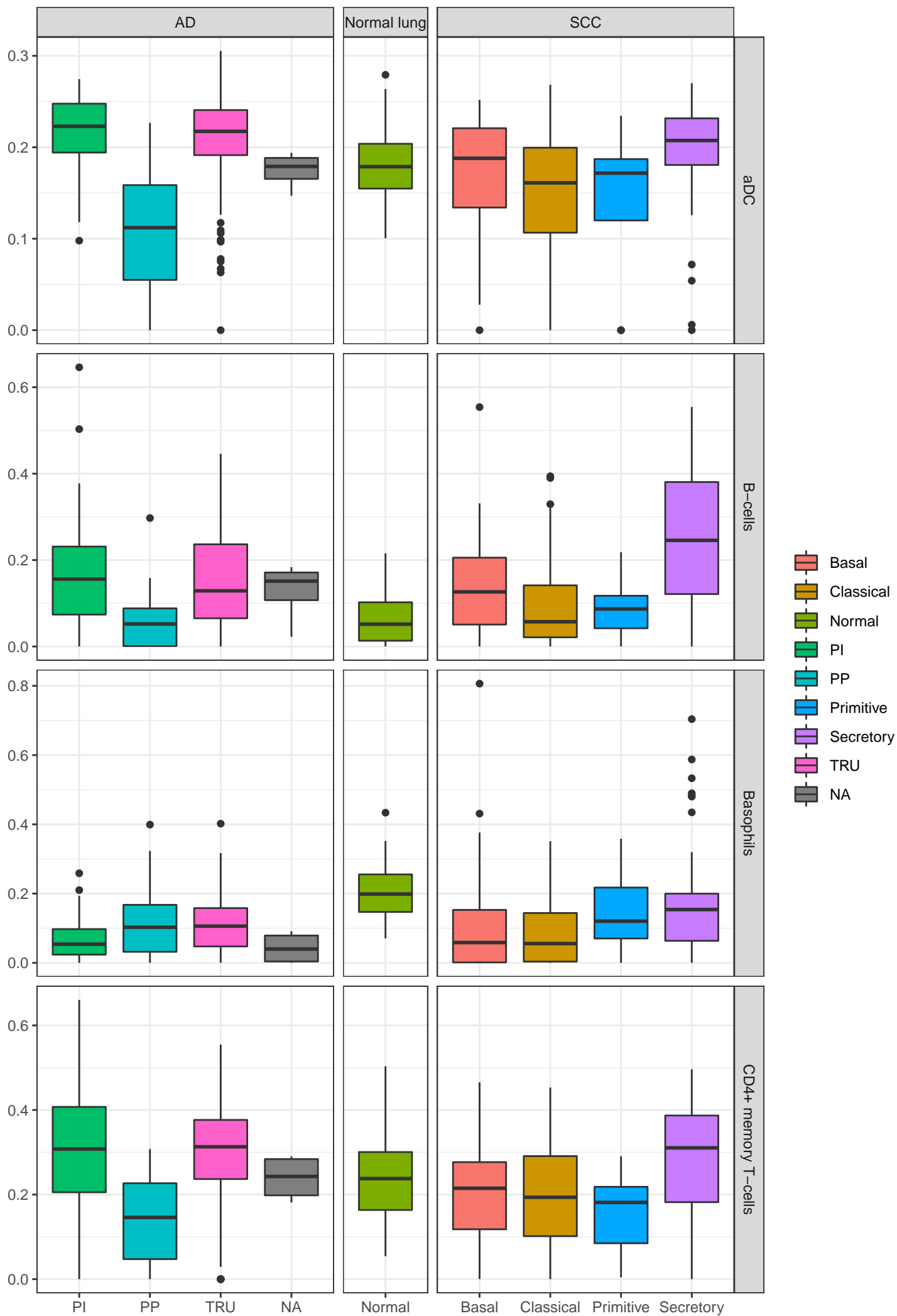

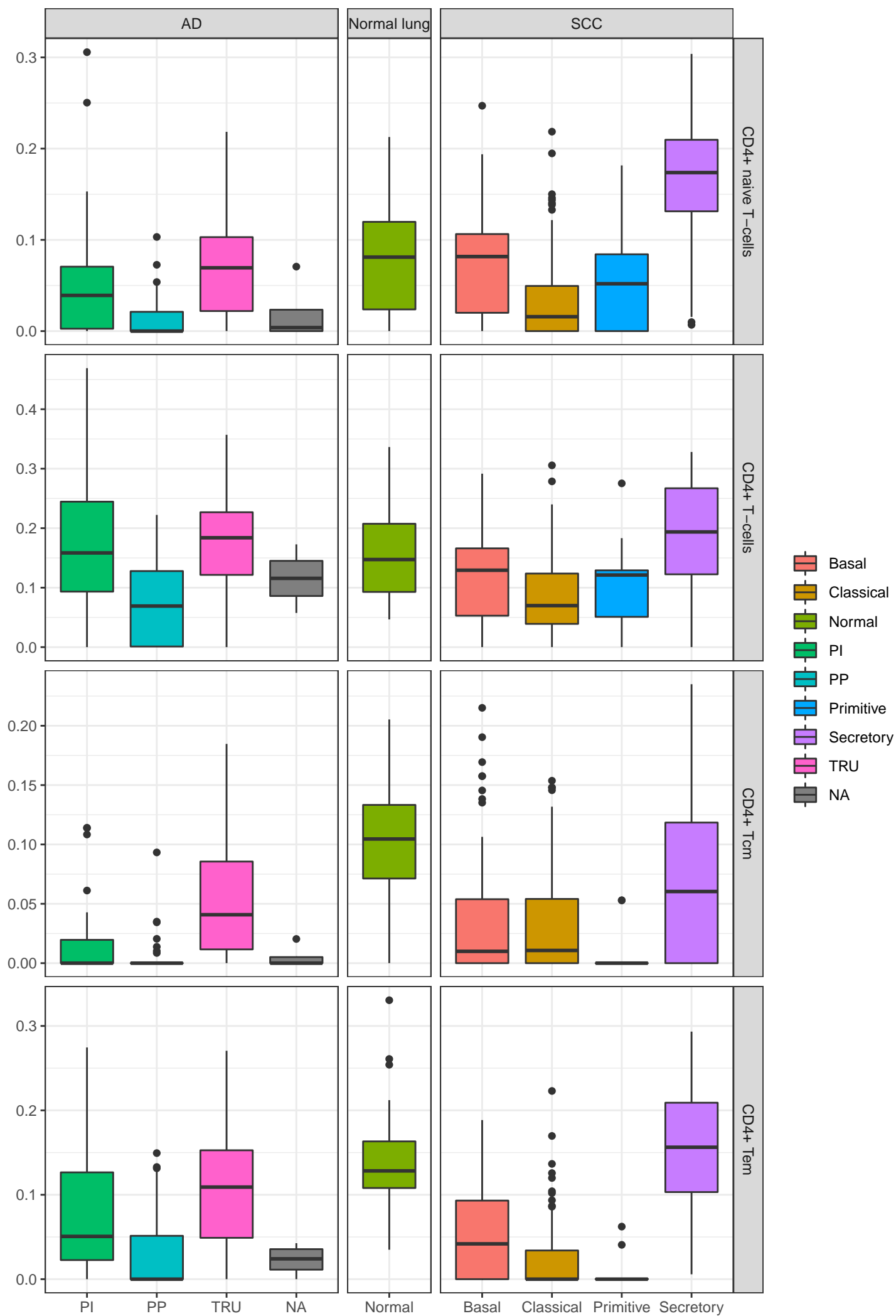

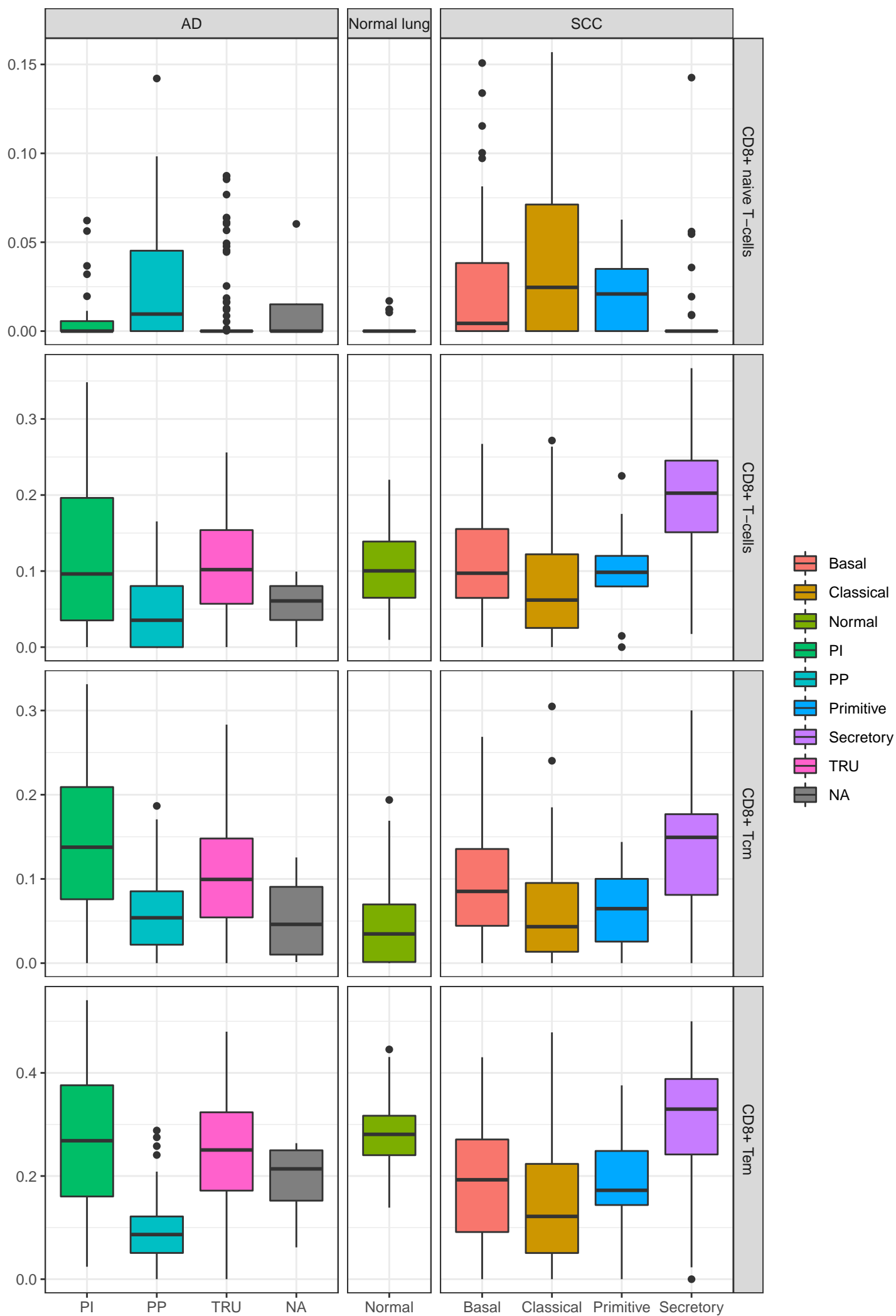

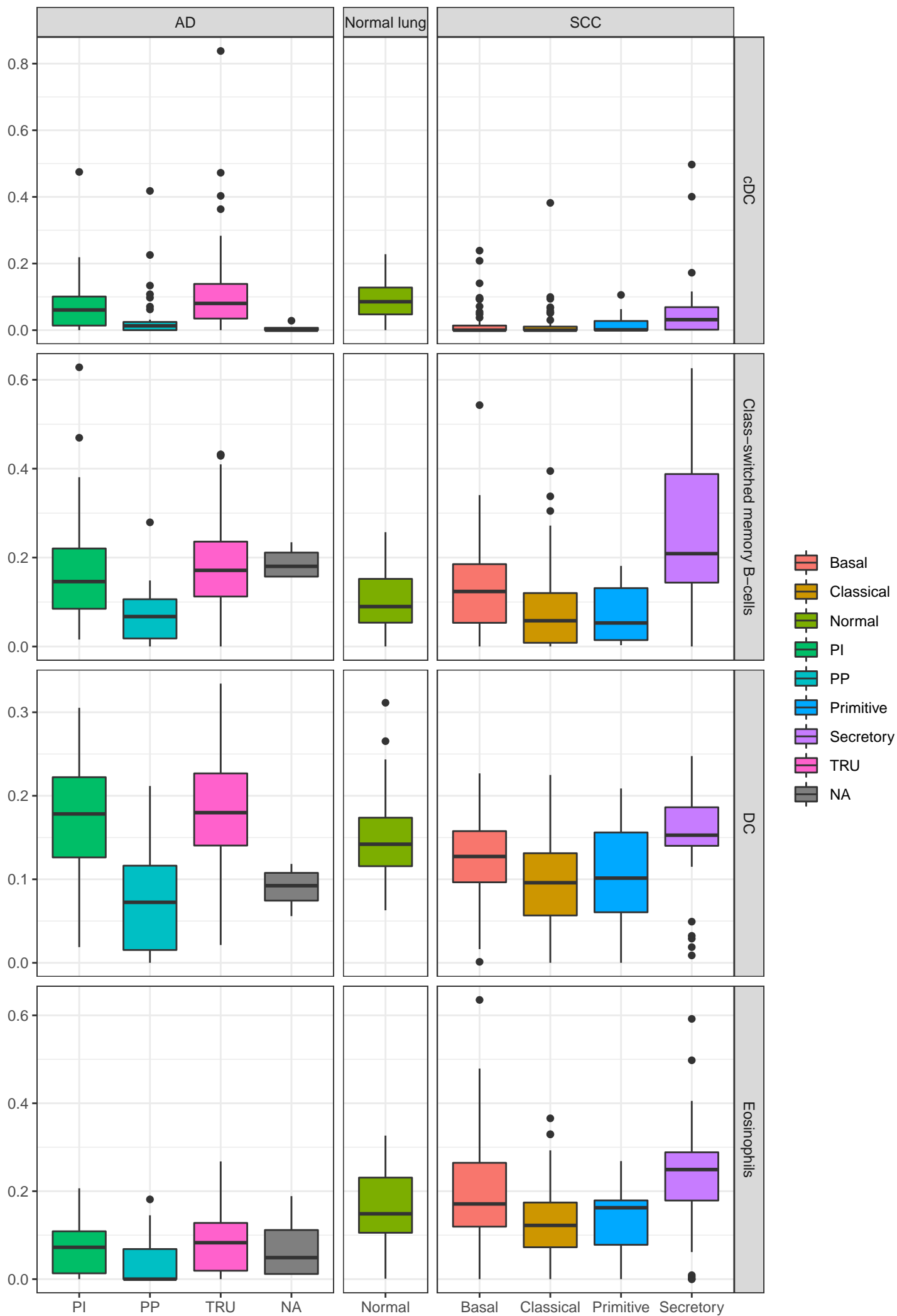

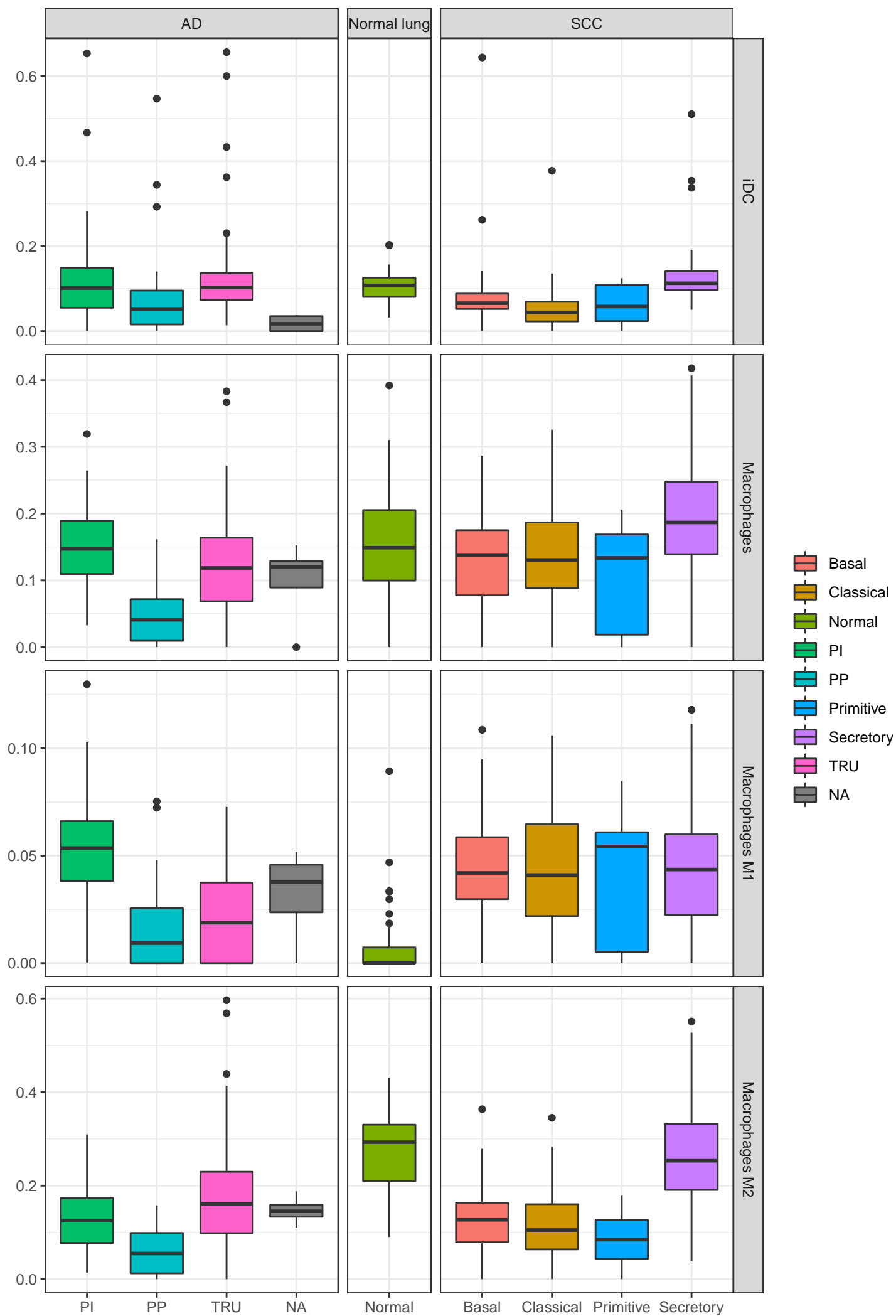

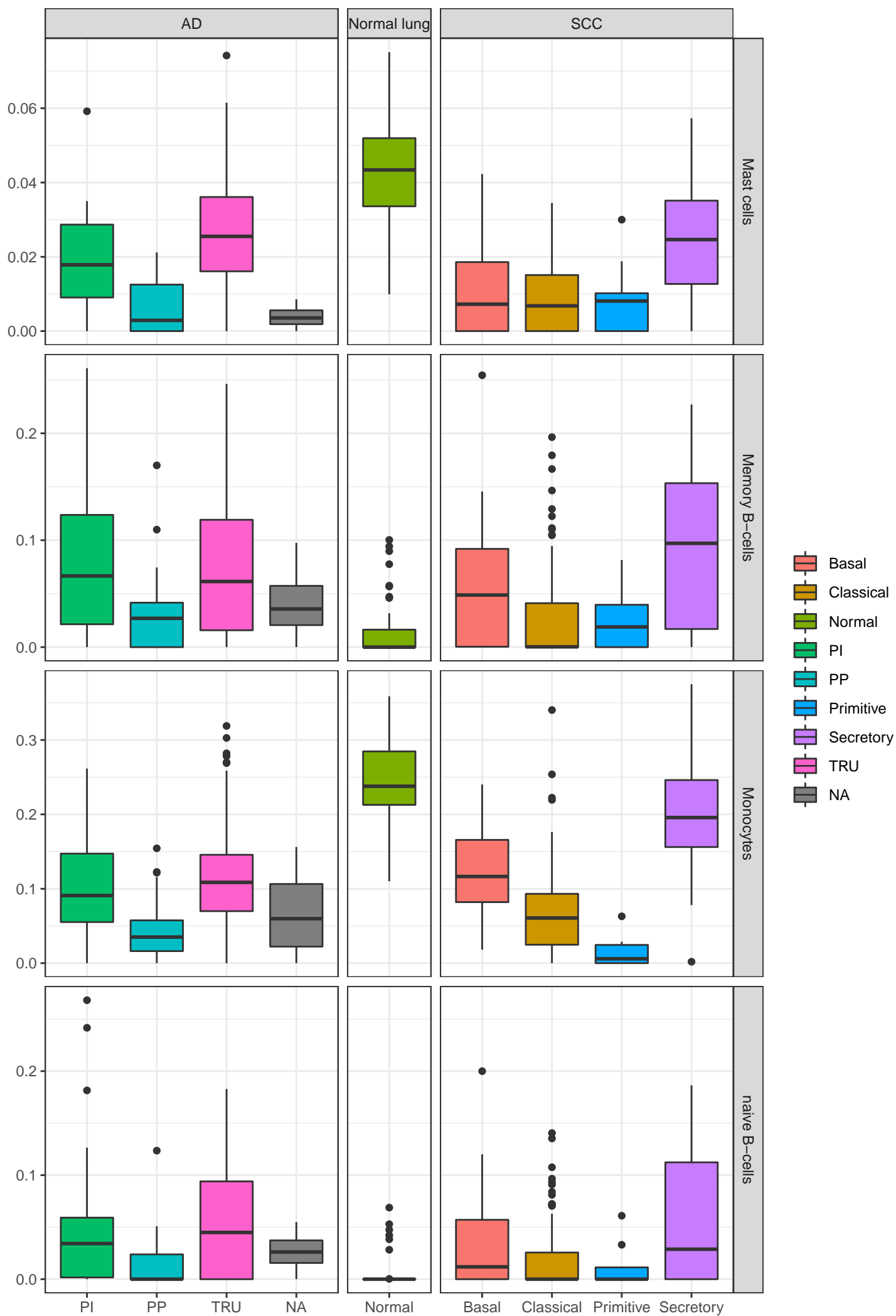

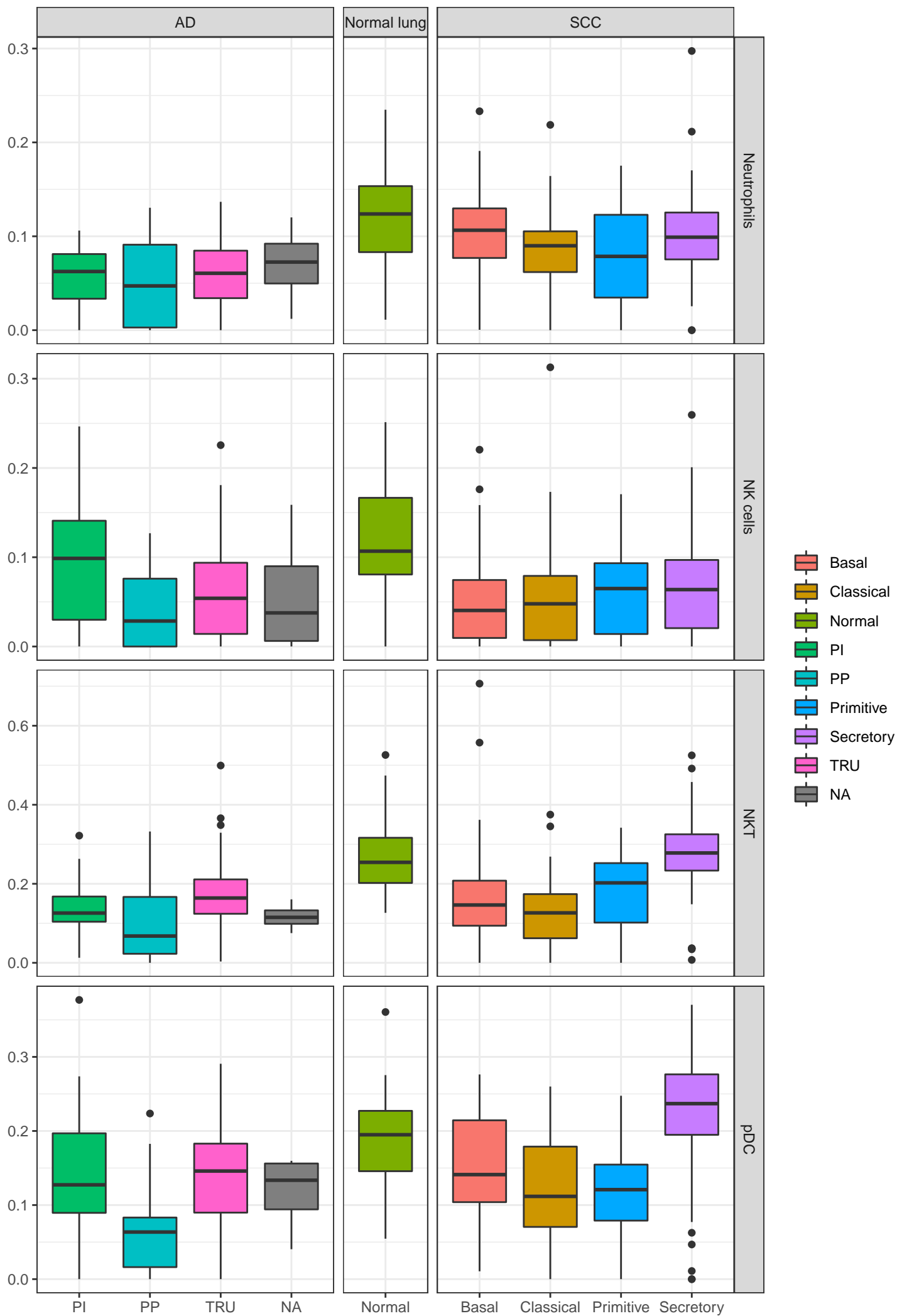

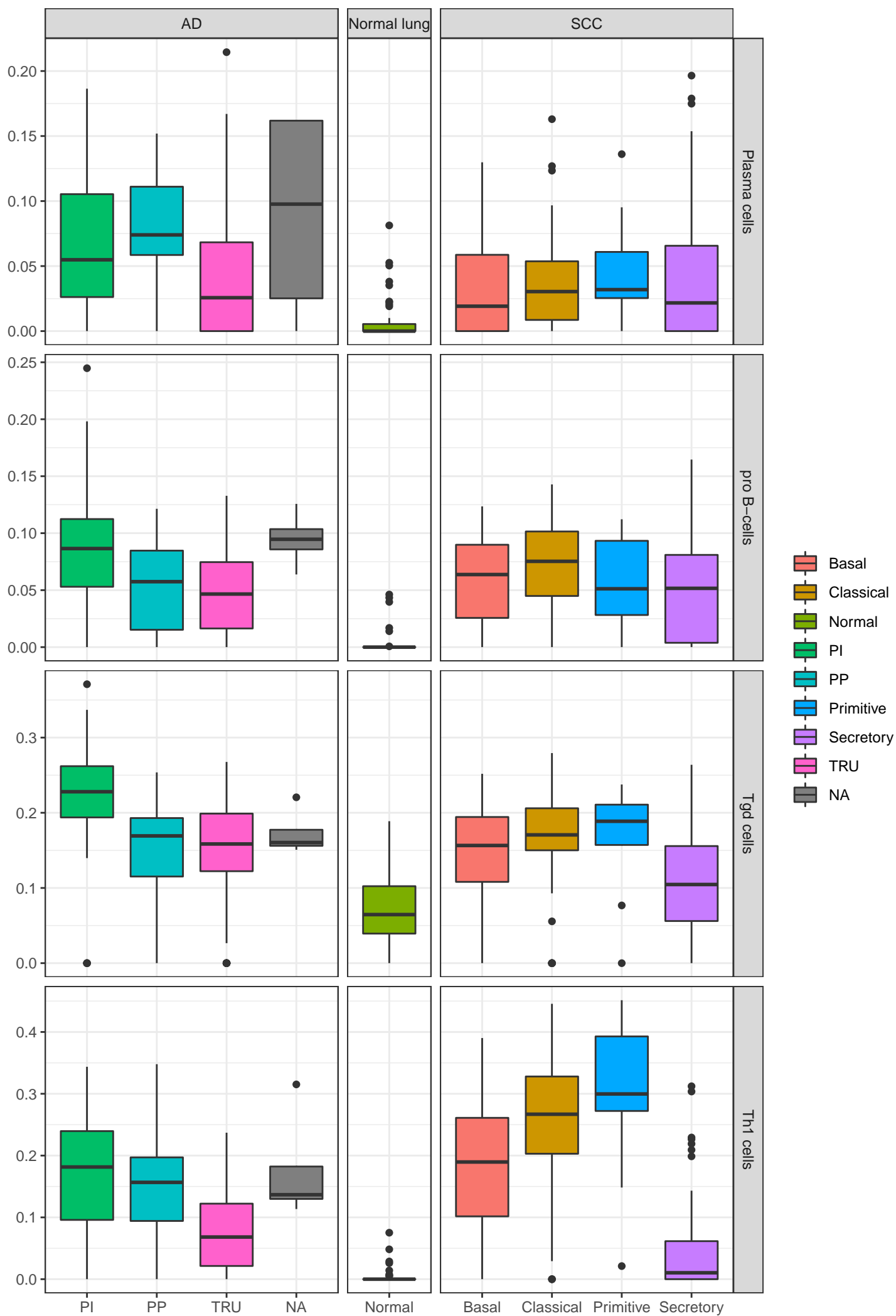

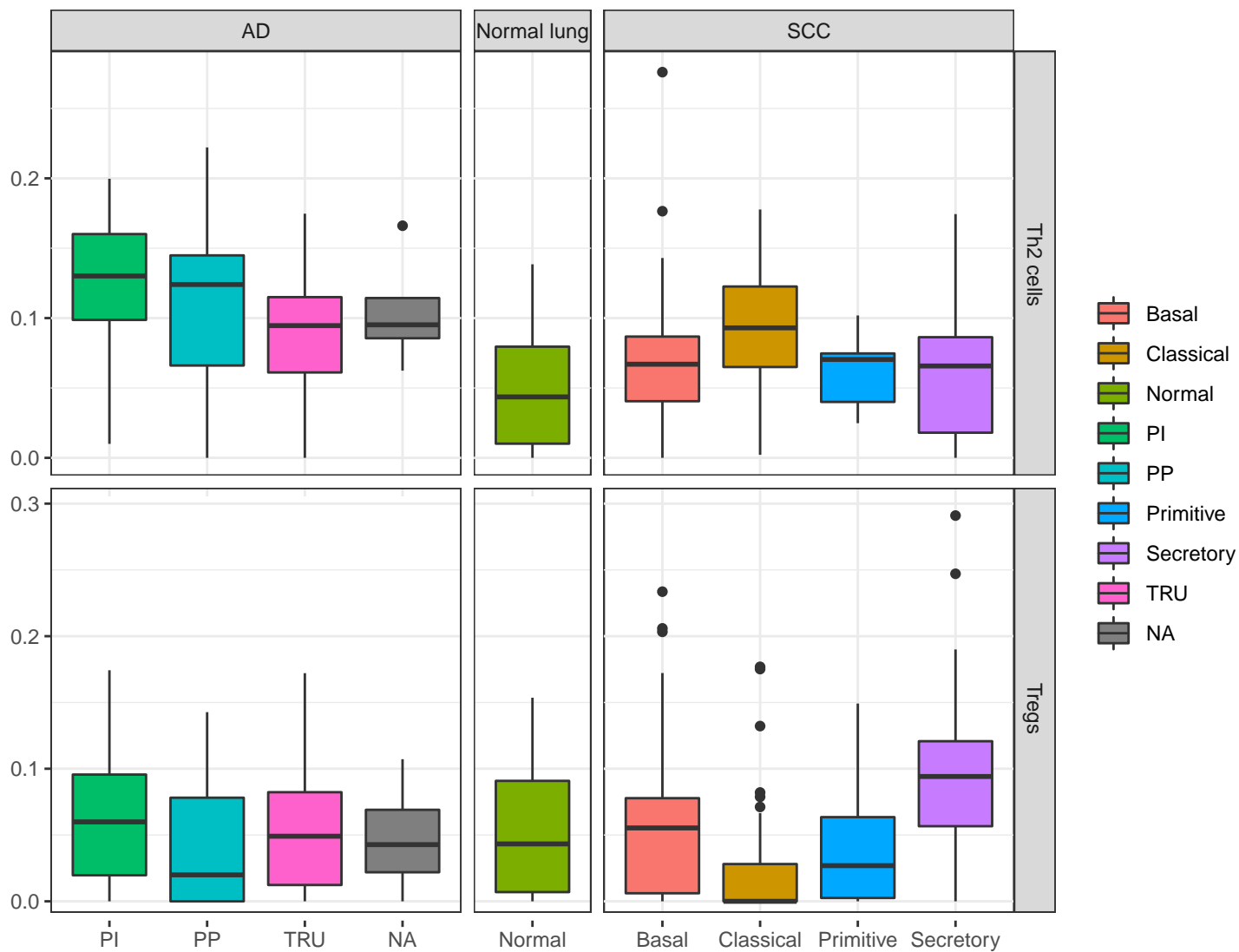

Supplement: Supplementary file 2 — Fig. S2. Box plots showing immune cell type estimates in adenocarcinoma and SCC expression subtypes and normal lung. [file MOL2-13-1166-s002.pdf]
