# Supplementary material for: The immune microenvironment in non‐small cell lung cancer is predictive of prognosis after surgery
Source: Mol Oncol. 2019 Apr 10;13(5):1166–79. doi: 10.1002/1878-0261.12475 (PMC6487716; doi:10.1002/1878-0261.12475)
Supplement: Supplementary file 3 — Table S1. Progression free survival analysis in adenocarcinoma expression subtypes. [file MOL2-13-1166-s003.docx]

|  | TRU | | PI | | PP | |
| --- | --- | --- | --- | --- | --- | --- |
|  | HR (95% CI) | P-value | HR (95% CI) | P-value | HR (95% CI) | P-value |
| TP53 mutation **  Wildtype or silent mutation  Non-silent mutation | 1  1.61 (0.87-2.99) | 0.132 | 1  1.13 (0.50-2.56) | 0.775 | 1  0.54 (0.22-1.35) | 0.191 |
| EGFR mutation **  Wildtype  Mutation | 1  1.27 (0.68-2.38) | 0.448 | _ | _ | _ | _ |
| KRAS mutation **  Wildtype  Mutation | 1  1.18 (0.63-2.19) | 0.604 | 1  2.69 (1.10-6.55) | 0.0299 * | 1  1.09 (0.36-3.29) | 0.884 |
| Immune score ** | 0.79 (0.58-1.08) | 0.138 | 0.71 (0.46-1.10) | 0.123 | 1.35 (0.68-2.71) | 0.394 |
| Cytolytic score ** | 0.98 (0.68-1.41) | 0.923 | 0.82 (0.56-1.22) | 0.337 | 0.64 (0.41-0.98) | 0.0415 * |
| Proliferation score ** | 1.08 (0.74-1.58) | 0.695 | 1.85 (0.91-3.76) | 0.0896 | 1.14 (0.66-1.99) | 0.636 |
| *CD274* gene expression ** | 1.23 (0.84-1.80) | 0.296 | 0.87 (0.56-1.34) | 0.524 | 0.70 (0.41-1.18) | 0.177 |
| IHC PD-L1 ** | 0.99 (0.70-1.39) | 0.94 | 1.01 (0.73-1.38) | 0.970 | 1.00 (0.60-1.66) | 0.990 |

Supplementary table 1. Progression free survival analysis in adenocarcinoma expression subtypes assessed by Cox proportional regression analysis. * *p < 0.05 ** Ajusted for stage.*
