# Supplementary material for: The immune microenvironment in non‐small cell lung cancer is predictive of prognosis after surgery
Source: Mol Oncol. 2019 Apr 10;13(5):1166–79. doi: 10.1002/1878-0261.12475 (PMC6487716; doi:10.1002/1878-0261.12475)
Supplement: Supplementary file 4 — Table S2. Progression free survival analysis in SCC expression subtypes. [file MOL2-13-1166-s004.docx]

|  | Basal | | Classical | | Primitive | | Secretory | |
| --- | --- | --- | --- | --- | --- | --- | --- | --- |
|  | HR (95% CI) | P-value | HR (95% CI) | P-value | HR (95% CI) | P-value | HR (95% CI) | P-value |
| TP53 mutation **  Wildtype or silent mutation  Non-silent mutation | 1  0.61 (0.17-2.20) | 0.446 | 1  1.94 (0.57-6.61) | 0.288 | _ | _ | 1  1.64 (0.45-5.96) | 0.455 |
| Immune score ** | 0.81 (0.43-1.53) | 0.521 | 1.35 (0.87-2.11) | 0.184 | 0.79 (0.24-2.59) | 0.692 | 0.98 (0.54-1.76) | 0.937 |
| Cytolytic score ** | 0.68 (0.39-1.18) | 0.170 | 1.10 (0.75-1.59) | 0.633 | 0.66 (0.18-2.50) | 0.546 | 0.66 (0.38-1.13) | 0.129 |
| Proliferation score ** | 0.71 (0.37-1.38) | 0.314 | 1.39 (0.67-1.89) | 0.382 | 4.76 (0.33-67.68) | 0.25 | 1.15 (0.62-2.13) | 0.653 |
| *CD274* gene expression ** | 0.75 (0.45-1.25) | 0.263 | 1.65 (1.18-2.31) | 0.00314 * | 1.0 (0.61-1.80) | 0.870 | 0.48 (0.24-0.97) | 0.0404 * |
| IHC PD-L1 ** | 0.58 (0.20-1.67) | 0.314 | 1.39 (1.01-1.90) | 0.0402 | 9.6e-01 (0.44-2.09) | 0.918 | 0.86 (0.48-1.52) | 0.596 |

Supplementary table 2. Progression free survival analysis in squamous cell carcinoma expression subtypes assessed by Cox proportional regression analysis. * *p < 0.05 ** Ajusted for stage.*
